# Developing a Dissemination Model to Improve Intervention Reach among West Virginia Youth Smokers

**DOI:** 10.3389/fpubh.2014.00101

**Published:** 2014-08-01

**Authors:** Kimberly Horn, Traci Jarrett, Andrew Anesetti-Rothermel, Nancy O’Hara Tompkins, Geri Dino

**Affiliations:** ^1^Department of Prevention and Community Health, Milken Institute School of Public Health, George Washington University, Washington, DC, USA; ^2^WV Prevention Research Center, School of Public Health, West Virginia University, Morgantown, WV, USA

**Keywords:** dissemination, dissemination science, diffusion, teen smoking, teen smoking cessation, tobacco prevention, tobacco intervention

## Abstract

The not-on-tobacco program is an evidence-based teen smoking cessation program adopted by the American Lung Association (ALA). Although widely disseminated nationally via ALA Master Trainers, in recent years, adoption and implementation of the N-O-T program in West Virginia (WV) has slowed. WV, unfortunately, has one of the highest smoking rates in the US. Although it is a goal of public health science, dissemination of evidence-based interventions is woefully understudied. The present manuscript reviews a theoretical model of dissemination of the not-on-tobacco program in WV. Based on social marketing, diffusion of innovations, and social cognitive theories, the nine-phase model incorporates elements of infrastructure development, accountability, training, delivery, incentives, and communication. The model components as well as preliminary lessons learned from initial implementation are discussed.

## Introduction

A core function of public health includes developing effective interventions that address priority health issues and assuring that those interventions are disseminated widely and sustained over time (1, 2). Although hundreds of public health interventions have been evaluated, a relatively small number have been proven effective. Moreover, only a handful of those proven effective are widely disseminated or “scaled up,” particularly for populations with the greatest health needs and disparities. Unfortunately, there is a dearth of research about and federal funding for dissemination research (3). A content analysis of 1,210 articles from 12 public health journals revealed that <1% of the articles addressed intervention diffusion (4). Other studies have estimated that <10% of prevention research is focused on dissemination (5, 6).

Attributing to this dearth of literature is the fact that little is known about effective strategies for dissemination and implementation of complex public health innovations across large health and education systems. Importantly, although dissemination research may be lacking, the public health practice literature identifies a number of impediments to the widespread dissemination of effective interventions. These include differences among delivery systems at state, regional, and local levels; the lack of a single, effective dissemination system; and insufficient organizational and personnel capacity (7). Other challenges include ineffective dissemination practices, lack of acceptability and buy-in, lack of feasibility, competing incentives, and external political or other forces. Taking all of these challenges into consideration, increasingly, state-level systems are essential to disseminating and supporting evidence-based interventions (2) to address public health priorities.

To that end, the need for high quality dissemination research is especially critical to address priority public health problems – *youth tobacco use is a chief example*. As one of the most costly public health issues in the United States, cigarette smoking (8) causes over 400,000 premature deaths and $157 billion in US health-related economic losses per year (9). Almost 4,000 youth initiate smoking each day and nearly one-quarter of US high school teens are current smokers (10). Over half of teens try smoking during their lifetime (11). Without effective intervention, most youth who smoke continue smoking into adulthood, elevating their lifetime risk for cardiovascular disease, several types of cancer (including lung cancer), and other debilitating conditions (12). Fortunately, 65% of daily teen smokers report that they want to quit (13, 14). The prevalence and consequences of teen smoking coupled with the new evidence on cessation program effectiveness, indicates an unequivocal need for effective, widely disseminated youth smoking cessation interventions (7, 15).

Kerner et al. (3) note, “efforts to move effective preventive strategies into widespread use too often have been unsystematic, uncoordinated, and insufficiently capitalized….” The purpose of this article is to describe a new theory-driven nine-phase dissemination model designed to expand the reach of a teen smoking cessation intervention called not-on-tobacco (N-O-T). N-O-T is a national program of the American Lung Association (ALA). In this article, we explain the rationale and conceptualization of the model, including the theoretical foundation, and the step-by-step operationalization of each of the nine phases, all within a real-word context. The challenges and lessons learned during the rollout of the model are discussed, and we chart the next steps of rigorous evaluation. The translation of this type of process is key to bridging the gap between science and practice (16, 17). We describe the application of the model using the state of West Virginia (WV) as a case example. Of importance, WV teens have among the highest smoking rates in the US, ranking 49th compared to all other states (11, 18, 19). Factors of cultural acceptance of cigarette use, rural communities and geographic isolation, and economic underdevelopment create strong counter forces to WV’s comprehensive statewide tobacco control programs (20). Although results from the West Virginia Youth Tobacco Survey from 2000 to 2011 indicate promising declines in youth ages 14–18 who report ever smoking cigarettes, down from 74% in 2000 to 50% in 2011 (19, 21), there remains a critical need to address prevention and cessation among teens in WV.

We chose WV to pilot the initial set-up and evaluation for the new dissemination model because of the long history of the program in the state (N-O-T was originally developed and tested in WV in 1998), the presence of a seasoned N-O-T ALA master trainer, and an established Community Partnership Board through the West Virginia Prevention Research Center (WV PRC) who can provide guidance and local support for the model. The nine-phase dissemination model builds on previous research by our team (22–25) and others consistently demonstrating that substantive change requires continuous technical assistance and accessible resources to guide users through the complex processes of dissemination (26–28). This model is informed by both the success and failures of previous efforts to disseminate evidence-based programs, including the N-O-T program (29–33). The aspects of this project that included the collection of human subjects data received IRB approval through West Virginia University (WVU).

## Not-On-Tobacco Program Overview

In 1998, West Virginia researchers and the WV PRC Community Partnership Board used community-based participatory research methods to develop a youth smoking cessation program designed for 14–19 year olds, called not-on-tobacco (N-O-T described in detail below). Following pilot testing in WV and multiple randomized trial and evaluation studies for effectiveness, the ALA adopted N-O-T as their premiere teen smoking cessation program, including a national train-the-trainer structure comprised of 10 N-O-T Master Trainers. This structure supports the implementation of a centralized dissemination model. It is currently the most used teen smoking cessation program in the nation (34) and has been incorporated into the WV Division of Tobacco Prevention comprehensive tobacco control efforts since 2000. However, even within the ALA’s national training infrastructure, lack of an evidence-based dissemination model impedes efficient and effective program monitoring, implementation, accessibility, and sustainability. Many states still experience barriers at various levels of implementation, including WV. Although N-O-T has a published evidence base and is available for widespread use, the program is currently not accessible to every teen who wants to use it. The magnitude of the tobacco use prevalence and the structure of tobacco control policy, and support for N-O-T cessation programing in WV present a unique opportunity to implement and evaluate a state-level model to disseminate youth tobacco cessation programing.

Extensively detailed elsewhere (24), the N-O-T core program consists of 10 50-min sessions that occur once a week for 10 consecutive weeks, with the option of 4 additional booster sessions. The core program is led by a trained facilitator, usually from within the teen’s school. Facilitators must be non- or former-smokers, be able to relate to teens, and be willing to work with school administration or community organization leadership to recruit teens and promote the program within their school or organization. They are also required to complete the N-O-T program’s 1 day American Lung Association’s Facilitator Training Workshop to ensure implementation fidelity. The interactive training provides a foundation for understanding the N-O-T program’s core curriculum and how to deliver it effectively. N-O-T facilitators are responsible for recruiting teens into the program, consisting of approximately 3–10 participants. As prescribed, teens are eligible for enrollment if they have smoked one or more cigarettes in the past 30 days, are interested in quitting, and volunteer to participate in the program.

N-O-T facilitators guide teens through each session utilizing a total health approach that focuses on healthy behaviors, stress management, and life skills. Specifically, the program includes motivational issues, smoking history, nicotine addiction, the physical, psychological, and social consequences of smoking, preparation for quitting, dealing with urges and cravings, relapse prevention, stress management, dealing with family/peer pressure, increasing healthy lifestyle behaviors in physical activity and nutrition, and volunteerism. Over a decade of published research shows that N-O-T is cost-effective (35), adoptable, and suitable for dissemination (36). N-O-T studies between 1998 and 2003 showed end-of-program intent-to-treat quit rates between 15 and 19% (25), among the highest rates reported in the literature (14).

## Existing Programing Challenges in WV

Consistent with our community-driven research approach, the investigators consulted with the WV Division of Tobacco Prevention to assess the challenges with the statewide reach of N-O-T. Subsequently, we conducted a systematic evaluation (37) to assess N-O-T barriers and successes to program implementation and dissemination in WV (36). Program evaluation data were analyzed geographically by eight Regional Education Service Agencies, called RESAs (38) representing 55 counties. N-O-T was offered inconsistently dispersed across WV’s 55 counties. Whereas there were 152 unique program offerings in 35 of the 55 WV counties (63.6%), 20 counties failed to provide N-O-T (39). Nineteen counties had trained facilitators but provided no N-O-T programs.

Interestingly, *every RESA* had at least 40 trained facilitators; about 700 facilitators were trained between 2000 and 2005 (39). Still, extrapolating from our WV public health and school health professionals who serve teens, we estimated that at least 84% of our potential facilitators had not been trained in N-O-T (40), reaching <16% of potential implementers. This assessment also helped us to understand our WV N-O-T participants. Our final N-O-T teen sample after excluding non-intervention programs (e.g., health classes), smokeless tobacco users, and non-smokers was *N* = 1,008 (39). N-O-T program enrollment increased sixfold during the evaluation time-period, with the highest number of participants (*n* = 246) in 2004 and the lowest in 2000 (*n* = 41) (39). The program enrolled slightly more females (53.8%) than males (46.2%) from 2000 to 2005 (39). The mean age was 15.8 (SD = 1.4). Teens smoked an average of 14.5 cigarettes on weekdays (SD = 13.2) and 20.5 on weekend days (SD = 20.7). Participants started smoking around age 10 (SD = 2.8). Overall, intent-to-treat analysis revealed 18.0% of teens had quit smoking. Furthermore, compliant-sub-sample analysis (*N* = 750) found that 24.1% of teens had quit smoking. Of the teens that did not quit, 67% reported smoking reduction. These quit rates are at or above national N-O-T averages, showing that WV efforts with N-O-T are positive when the program is offered. Unfortunately, our findings suggest that even in the year of our highest reach (2004), given a 29% smoking rate in WV’s 14–18 year olds), N-O-T’s overall reach among WV teen smokers was <1%.

Previous N-O-T studies identified dissemination barriers at facilitator and teen participant levels (36, 41). In WV, in particular, some facilitators never attempted use, whereas others attempted but could not successfully recruit. Facilitators reported that there were no systematic mechanisms in place to garner administrative support, reinforce and monitor program delivery, or help with recruitment. Findings reinforced our assumptions about low implementation following training and differences by region. This work, along with extensive discussion with community and state partners, generated a list of specific barriers to address in our model: (1) lack of uniform decision making and consistent buy-in across stakeholders, (2) inconsistent communication and understanding of the impediments to dissemination, (3) uneven distribution of labor (one person cannot do all that is needed for effective dissemination), (4) non-systematic program promotion, (5) low compliance following training, recruitment, and retention issues among N-O-T teens, (6) no uniform assignment of facilitator monitoring, (7) inconsistent and non-compliance with reporting, (8) low to no incentives at regional, school, or site levels, and (9) lack of persistent technical assistance. The dissemination model seeks to address these barriers theoretically guided by Diffusion Theory and Social Cognitive Theory.

## Dissemination Model Conceptualization

### Theoretical framework

*Diffusion Theory* explains the patterns of adoption of an innovation by individuals and organizations over time and critical attributes that influence adoption. Rogers (40) illustrates an *adoption pattern* that is depicted by an S-shaped curve, characterizing individuals/organizations as innovators, early adopters, early majority, late majority, or laggards, based on when they adopt an intervention relative to others on the curve. Given this S-curve, our ongoing assessments suggest that N-O-T has entered the initial process of adoption (innovators, early adopters) but has not yet reached the majority of its potential users in WV. We assert that a centralized model of dissemination will enhance the dissemination of N-O-T, particularly in the areas of reach, adoption, and implementation. In the context of our WV case study, N-O-T is perceived as an improvement over existing practice (relative advantage); is perceived as easy to use (complexity); is perceived as consistent/compatible with existing values and practices (compatibility); can be modified and still be effective (flexibility); and has observable results (observability). Diffusion Theory also describes five distinct steps that characterize an *implementer’s decision to adopt:* (a) gains understanding of how the intervention works (knowledge); (b) forms a favorable attitude about the intervention (persuasion); (c) engages in activities that lead to a choice to adopt (decision); (d) utilizes the intervention (implementation); and (e) seeks reinforcement for implementation (confirmation). These steps are woven throughout the nine phases of the model, with the goal of continually moving stakeholders and users to adopt, implement, and sustain N-O-T.

*Social Cognitive Theory* was used to address the individual decisions involved in model dissemination that could not be thoroughly addressed by the systematic institutional framework of Diffusion Theory. Thus, to ensure that our model also addresses the importance of individual behavior, key social cognitive constructs include modeling and incentives. According to Bandura (42), modeling is a key way for learning about an innovation. Modeling can occur through direct observation or through symbolic modeling (e.g., media). Modeling can inform potential adopters about the positive attributes N-O-T and can provide reinforcement for adoption, implementation, and maintenance. Incentives also play a major role in influencing behavior and can be used to reinforce initial and continued use of N-O-T. Different types of incentives may include social, monetary, status and power, and self-evaluative incentives.

Finally, we apply the elements of *social marketing* to complement the theoretical frameworks at both the institutional and individual levels. Three social marketing approaches intended to facilitate adoption and implementation are built into the components of the model: (1) conducting formative research with prospective adopters (e.g., regions, schools, facilitators) to understand how an evidence-based intervention (i.e., N-O-T) can promote stakeholder missions and increase the likelihood of adoption; (2) developing sustainable channels (i.e., regional networks) to promote and implement N-O-T; and (3) improving intervention access (e.g., widespread N-O-T implementation). We also incorporate the four Ps of social marketing – product at a minimal price; maximize the places that offer N-O-T programs using regional delivery; and promote program by multiple channels at multiple levels.

## Dissemination Model Description

**Figure 1:**
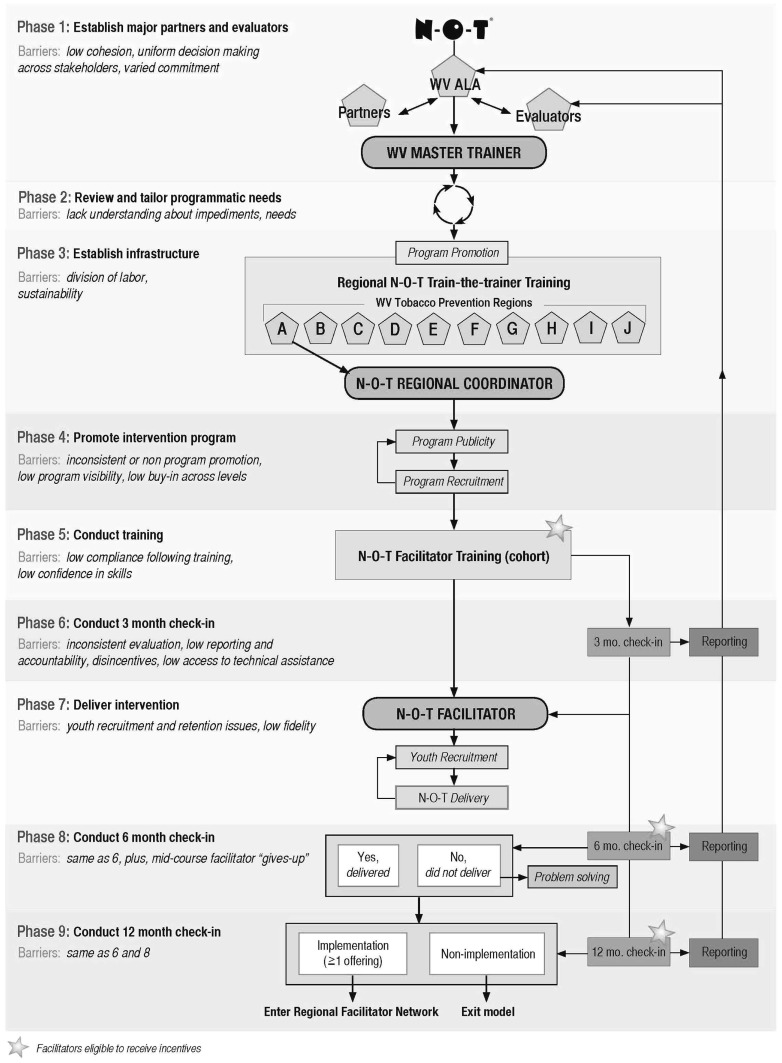
**N-O-T dissemination model**.

The N-O-T Dissemination Model is presented in Figure 1. The starting point of this model is based on three assumptions. *Assumption 1*: an initiator/leadership group for a given community or target population has identified teen smoking cessation as a health priority requiring intervention. *Assumption 2*: N-O-T has been selected as the evidence-based intervention to address the priority. *Assumption 3*: the initiator or leadership group has established contact with a local ALA and has (a) confirmed N-O-T availability and (b) has permission to proceed with a widespread dissemination.

## Elements of the Model

Consistent with theory and our preliminary work, the model incorporates eight essential elements: (1) *Infrastructure*: interconnected services, facilities, and resources that are often fixed or permanent in a given state/region and are necessary for N-O-T dissemination. Infrastructure can include individuals but must be more than individuals. In WV, the major infrastructure is our *10 Tobacco Prevention Regions*. (2) *Implementers:* individuals in a given locale who are responsible for completing assigned specific tasks relevant to implementation. Key implementers are trained, qualified persons who enable implementation by assisting with communication, linking systems, channeling resources; and acting as role model, mentor, or expert. Implementers also encourage expression of feelings and opinions, gather and dispense information, provide support, and administer incentives. *Key implementers include the N-O-T Master Trainers, Regional Coordinators, and N-O-T facilitators*. (3) *Task accountability:* all participants in the dissemination process have specified functions and tasks throughout. Each implementer is provided a *Task List* outlining responsibilities and is asked to sign a *Memorandum of Understanding*. In addition, step-by-step protocols for model implementation are provided to Master Trainers and Regional Coordinators as a part of training. (4) *Training:* the acquisition of knowledge, skills, and competencies to deliver the N-O-T training (train-the-trainer) and to deliver N-O-T (facilitator training). The ALA has *standardized training protocols* for both training levels. (5) *Critical assessment:* provides formative information to promote N-O-T implementation. Regional Coordinators provide multiple check-ins with facilitators following training to determine ongoing activity and levels of implementation since training. Feedback, although repetitive, is short term and intended to move non-implementing facilitators toward implementation and maintain the motivation of active implementers. (6) *Intervention delivery:* implementation of N-O*-*T as intended. (7) *Incentives:* consistent with Social Cognitive Theory successful dissemination requires that incentives be integrated across all model phases and be valued by recipients in order to enhance motivation. In our model, forthcoming incentives are introduced during trainings and at check-ins to motivate action and raise the expectations for reward. We employ three types of incentives: *social, monetary/material, and status/power*. Incentives occur at four levels within the model: (a) administratively (from the WVALA to the Master Trainer); (b) regionally (from the Master Trainer to the Regional Coordinator); (c) among users (from the Regional Coordinator to the Facilitator); and (d) among the targets (from the Facilitator to the Teens). (8) *Communication:* consistent with Rogers’ theory of diffusion, communication is necessary to create and share information across users, and participants to promote mutual understanding. Social Cognitive Theory also posits that individuals need consistent feedback to maintain desired behaviors. We integrate *formal* (i.e., fixed and required) and *informal* (spontaneous and as needed) communication. One of the model’s most significant communications occurs between the *Regional Coordinators and the* Facilitators (described in Phases 6, 8, and 9 below).

## Operationalizing the Dissemination Model Phases

### Phase 1: Establish major partners and evaluators

Phase 1 involves engaging major partners who are key to program uptake, implementation, and sustainability. However, it does not preclude the involvement of other partners. Major partners should be represented by one or more individuals who are key decision makers or who have immediate access to key decision makers. As described above, diffusion experts contend that it is critical to obtain early buy-in from these stakeholders by illustrating how the program or intervention: (a) is viewed as relevant and meaningful to key decision makers and their organizations and how it is compatible with organizational goals; (b) can assist the organization in meeting programmatic objectives, also compatibility; (c) is cost-effective and consistent with evidence-based guidelines, complexity, and relative advantage; and (d) provides feedback mechanisms to support a decision to sustain the intervention over time, which will increase observability (40, 43–46). Clarifying needs and goals of major stakeholders helps to shape understanding of what incentives will be effective at an organizational level (in line with Social Cognitive Theory), as well as the four Ps of social marketing that make a “product” that appeals to a growing and evolving audience. According to Mailbach (45), major stakeholder organizations are essential for “building sustainable distribution channels to promote and deliver evidence-based programs to prospective adopters” (p. 1). From its inception, researchers worked with major partners including the Community Partnership Board of the WV PRC, the American Lung Association in West Virginia (WV ALA), the WV Department of Education, and the WV Division of Tobacco Prevention. These partnerships, which allowed for the identification of an issue critical to WV youth (cigarette use) through the WV PRC Community Partnership Board, and the development of N-O-T, including its dissemination in WV schools, allowed for re-assessment and identification of challenges over time as described in Phase 2. Major partners were a part of decision making prior to Phase 1, inclusive of the selection of an evaluator (43). Key evaluative components may include assessment of intended/unintended consequences of N-O-T, cost-effectiveness, and impact on participant and organizational outcomes. An established evaluation unit or provider is ideal.

### Phase 2: Review and tailor programmatic needs

Dissemination can be described along a continuum from no adoption to full adoption (including stakeholder buy-in, Regional Coordinator and facilitator training, teen recruitment, implementation, and reporting). When major partners come together with the intent to disseminate an intervention such as N-O-T, it is usually under one of several circumstances. First, they have never attempted to disseminate N-O-T broadly and want to determine the most effective strategy. Second, they have attempted some type of dissemination but uptake and adoption have had varied degrees of success in different settings and with different sites. Third, all aspects are successful. In WV, the overwhelming majority of regions had experienced the first two scenarios. As such, it is necessary to conduct a collaborative needs assessment to determine the potential dissemination impediments or catalysts, particularly as they relate to infrastructure. Notably, we had already conducted our needs assessment, previously described in the background section of this article. As discussed earlier, our preliminary data suggested that the tasks of dissemination needed to be evenly distributed across the state, without the full burden of dissemination on the single WV ALA N-O-T Master Trainer. The intended effect is a reduction in complexity of facilitator recruitment, training, reporting, and continued support, provide flexibility in program delivery, and offer an advantage in barrier reduction under the current model. In order to reduce burden on the single delivery of N-O-T facilitator training, Phase 3 addresses identification and establishment of an infrastructure within which to enhance program delivery.

### Phase 3: Establish infrastructure

We define infrastructure as interconnected services, facilities, and resources that are often fixed or permanent in a given state or geographic location and are necessary to support and disseminate a particular intervention. Mailbach (45) refers to this concept as “distribution channels.” Sometimes infrastructure involves or is a part of the major partners identified in Phase 1, sometimes it is not. Our previous state-level research with N-O-T in two very different states, WV and FL (23, 25), strongly indicate that a clearly identified infrastructure or network of distribution channels is necessary for the effective dissemination and long-term program sustainability. In fact, infrastructure is the “tipping point” of an intervention’s success or failure with widespread dissemination. Division of dissemination labor is an important part of dissemination infrastructure (47). To maximize infrastructure for N-O-T dissemination, our model divides labor across three levels. (a) *State-level major partner infrastructure:* our current project engages the infrastructures of our major partners who provide critical assets. The WV Department of Education provides access to schools and other sites that serve teens and the Regional Coordinators, whose salaries they support; the WV Division of Tobacco Prevention provides access to expert staff; and WV ALA, provides access to a N-O-T Master Trainer, as well as other assets. (b) *Regional infrastructure:* often, the division of dissemination labor stops with the major partners and is assigned to one or two major partners, or even worse, one or two individuals or implementers within a state. As we have learned in WV, this strategy makes it impossible to disseminate an intervention broadly. Thus, a next step is to select a *regional infrastructure*. Our model proposes a regional system that equally divides dissemination labor and provides primary points of contact across the five selected treatment regions. We engaged long-time partners at the Division of Tobacco Prevention who had an existing infrastructure of Regional Tobacco Prevention Specialists (RTPS) throughout the state to act as our points of contact (i.e., Regional Coordinators) in each region. However, prior to the implementation of the proposed dissemination model, the RTPS network dissolved and in order to test the model, we needed to find a viable alternative delivery method. Fortunately, we were able to establish a new partnership with WVU Extension Service 4-H Youth Development Program agents to serve as the Regional Coordinators for the project. (c) *Site-level infrastructure*. Site refers to the place or location of actual N-O-T delivery (e.g., a school or community center). Sites provide necessary implementation assets such as meeting rooms, mechanism to recruit teens, and access to facilitators. Disincentives must be removed. However, it is important to give careful consideration to the model’s impact on current duties and workload. Once an infrastructure is identified and developed, Regional Coordinators use their knowledge and connections to promote the program and train facilitators.

### Phase 4: Promote intervention program

Dissemination and marketing theories align with relative advantage of Diffusion of Innovations theory and suggest that dissemination of a public health program is maximized when potential adopters are: (a) aware of a public health need, (b) are aware of evidence-based, cost-effective approaches to address that need, (c) perceive that a particular program has advantages over other options for addressing the need; (d) perceive organizational and target population benefits to program adoption where benefits outweigh costs, and (e) have the capacity to implement the intervention (43, 47, 48). These factors can be addressed using social marketing approaches to program promotion (46, 49). We define “Promotion” as the *advertising, publicity, and personal selling* of N-O-T by our major partners generally and the key implementers, specifically the Master Trainer, the Regional Coordinators, and facilitators. The model relies on principles and practices from Social Cognitive Theory, Diffusion Theory, and social marketing approaches to develop and utilize promotional concepts and techniques designed to address specific behavior change goals at multiple levels of the dissemination process. Social marketing includes the application of commercial marketing techniques to the design, implementation, and evaluation of programs to promote socially positive behavior change (46, 50). For instance, N-O-T dissemination strategies incorporate promotion at site (organizational) and individual (facilitator, participant) levels; our distribution channels consist of individuals (Regional Coordinators, sites, facilitators) and organizations (schools, alternative schools, community centers). Consistent with Diffusion Theory, program promotion is intended to increase stakeholder: (a) awareness of N-O-T, (b) perceptions that N-O-T is a cost-effective way to address personal and organizational teen smoking cessation goals, (c) beliefs that N-O-T has advantages over other options, (d) decisions of adoption; and (e) commitment for implementation and long-term use. The type of promotion is about “the social good” rather than “financial gain,” although considerations of costs are relevant. Importantly, task accountability is essential in this phase as the key implementers have assigned tasks for promotion and training. Specifically, as shown in Figure 1, the Master Trainer is responsible for program promotion and Regional Coordinator training. In turn, the Regional Coordinators are responsible for program publicity, facilitator recruitment and training, and monitoring. Consistent with our noted theories, the promotional efforts of the Master Trainer and Regional Coordinators are guided by a four-point message (51): *Product*: N-O-T is evidence-based, cost-effective, includes national and local level support networks, is easy to implement, and better than the competition; *Price:* benefits of using N-O-T (promotion of teen health, reduction of tobacco violations, addressing state-mandated tobacco control goals) outweigh costs (time, resources). Incentives for program adoption and implementation are provided by state-level infrastructure; *Place:* N-O-T facilitator training and N-O-T delivery occurs locally (is accessible); *Promotional materials*: N-O-T provides ready materials including testimonials, brochures, flyers, PSA/media announcements, recruitment posters, and practitioner-friendly summaries of evidence-basis. Once program promotion is underway, Regional Coordinators with assistance from the State Coordinator can conduct training with potential N-O-T facilitators. In accordance with the RE-AIM framework, the number of potential implementation sites in this phase is used to determine the denominator of the program reach.

### Phase 5: Conduct training

All of our trainings follow standardized protocols. During training, all implementers receive their Task Lists, Memorandum of Understanding, and Step-by-Step Protocols for Delivery. *Train-the-Coordinator:* in the case study, the WV ALA N-O-T Master Trainer trains all five Regional Coordinators on model implementation with supplemental training on the roles and responsibilities of the Regional Coordinators, communication, and documentation conducted by research team staff. Training occurs over 2 days, including the regular N-O-T facilitator training and an intensive training on Regional Coordinator responsibilities. *Facilitator training*: for our study, the Regional Coordinators conduct facilitator training according to ALA guidelines with additional information about research protocols at the end of the training. Facilitators who are interested in participation in the study complete an informed consent document related to the enhanced communication schedule and human subjects’ protection. Per ALA N-O-T protocol, facilitators receive a copy of the curriculum, and didactic and experiential instruction on the N-O-T curriculum. Training lines up with the five distinct steps of adoption described in Diffusion of Innovations theory: knowledge, persuasion, decision, implementation, and confirmation. Training also applies knowledge gained in Phase 2 to identify appropriate incentives (Social Cognitive Theory) to induce adoption and maintenance. *Training also is tailored to address some of the key issues identified in our needs assessment, including negotiating with administration and other school personnel regarding time and resources, and teen recruitment*. It is important to hold trainings at a place of convenience, as determined by our partners. All trainings are evaluated, per N-O-T protocol. In order to assess whether the issues identified in the needs assessment are being adequately addressed, we established set check-ins throughout the entire adoption process including, N-O-T teen recruitment and implementation. This allows for quick response and flexibility if a trained facilitator reports a barrier at any stage of adoption and implementation. The number of facilitators trained in Phase 5 serves as the numerator for determining reach.

### Phase 6: Conduct 3 month check-in

Regional Coordinators have task accountability for checking in with N-O-T facilitators within 3 months following N-O-T training. Monitoring: Regional Coordinators monitor facilitators by collecting process and outcome evaluation data, with emphasis on implementation impediments/enhancers and teen recruitment. It is expected that at 3 months post training facilitators should have either begun implementation of their first N-O-T offering or begun planning for implementation. Standard data collection tools are used for Check-ins. Key Check-in queries include: has a N-O-T offering been scheduled, what recruitment methods are being used, what barriers/enhancers have been encountered, what potential solutions have been identified? N-O-T facilitators have the option of accessing the reporting forms electronically (via the Internet) or through the mailed survey forms (sent by the Regional Coordinator). Reporting: normally, the Regional Coordinators are responsible for collecting data and reporting findings to the ALA and the evaluators. In our study, the WVU research team works with the Regional Coordinators to sort and analyze the collected data as part of each Check-in. Mentoring: mentoring reflects the data collected through monitoring. Regional Coordinators provide positive feedback or praise to the facilitators who have either begun a N-O-T program or have one scheduled. Regional Coordinators also reinforce implementation of the standard N-O-T curriculum and assist with any necessary tailoring for N-O-T given their site needs. The Regional Coordinator facilitates problem solving with the cohort of facilitators through a Listserv and links facilitators to other facilitators in or outside of the cohort. Note: mentoring is a form of communication that is a crosscutting essential model element; thus, it is on going (informal) as well as during check-ins (formal). Incentives: all newly trained Facilitators receive a certificate of completion from the Regional Coordinators. At this point, the incentives are at social and status levels. The incentives for the 6-month Check-in are introduced at this time to motivate facilitators to action.

### Phase 7: Deliver intervention

This model intends that N-O-T is delivered as prescribed in the N-O-T curriculum. Facilitators have the task accountability for recruiting youth and delivering the program. It is expected that each facilitator implement >1 N-O-T offering within 6 months of training; >2 within 12 months. The N-O-T curriculum provides extensive detail on recruitment and implementation; however, Regional Coordinators assist facilitators with any implementation challenges during Check-ins. The sites for implementation are linked to the trained N-O-T facilitators – in WV, there are over 300 potential sites across regions. To address recruitment and other barriers identified in our preliminary studies, our goal is to provide ongoing mentoring and technical assistance to facilitators, enhance training on recruitment, and tailor promotional materials. Adoption and implementation are in accordance with the assessed as the number of N-O-T programs successfully offered to teens.

### Phase 8: Conduct 6 month check-in

Monitoring: as in the 3-month Check-in, Regional Coordinators monitor facilitators by collecting process and outcome evaluation data, impediments to implementation, and youth recruitment. It is expected at 6 months post training that N-O-T facilitators should have completed one N-O-T offering. The 6-month Check-in includes a Critical Assessment (40, 52) that helps the Regional Coordinator determine if facilitators delivered the program as scheduled. Other queries include: was the N-O-T protocol followed, what barriers/enhancers were encountered, how many teens enrolled, how many teens attended/completed N-O-T, what quit/reduction outcomes were found? Again, facilitators may access the reporting form electronically or through the mail. Regional Coordinators engage in *problem solving* with non-implementers. Reporting: (see Phase 6). Mentoring: coordinators provide positive feedback or praise to the facilitators who completed a N-O-T offering; coordinators troubleshoot with those who did not. Incentives: facilitators who completed a N-O-T offering receive a monetary incentive of $250 (paid by the ALA). They also receive a certificate of completion. Facilitators who complete >1 N-O-T offering within 6 months are awarded a “bronze” status, a way of recognizing experienced N-O-T facilitators.

### Phase 9: Conduct facilitator 12 month check-in

Monitoring: as in the 3- and 6-month post training check-ins, Regional Coordinators monitor N-O-T facilitators by collecting process and outcome evaluation data, with emphasis on impediments to implementation and recruitment. It is expected that N-O-T facilitators have, at the very least, completed one N-O-T offering. Key queries include: did they implement the program, did they follow protocol, what barriers did they encounter, how many teens enrolled, how many teens attended or completed N-O-T, what quit or reduction outcomes were found? Facilitators are also asked for input on program improvements. Again, facilitators may access reporting forms electronically or through the mail. The 12-month check-in is a Critical Assessment to identify if the facilitator has conducted a N-O-T group. Again, Regional Coordinators engage in *problem solving* with non-implementers. Reporting: in the proposed study, the WVU research team assists the Regional Coordinators with collecting the data and analyzing the findings. Mentoring: Regional Coordinators provide positive feedback to the facilitators who have completed a N-O-T program. The Regional Coordinator encourages non-implementers to offer N-O-T within 3 months and assists with troubleshooting. The Regional Coordinator continues to reinforce the ALA’s implementation protocol. At the end of Phase 9, the cohort of active facilitators that trained together is linked to the regional N-O-T facilitator network for future support and communications. Facilitators who have not implemented a N-O-T offering exit out of the model and are not be included in the regional network. Incentives: facilitators who complete a N-O-T program receive a monetary incentive of $250. In addition, they receive a certificate of completion. Continuing from the 6-month Check-in, Facilitators who complete >1 offering receive “bronze” status. Those who complete the N-O-T program >2 times receive “silver” status. Facilitators who complete >4 receive “gold” status.

## Next Steps and Preliminary Lessons Learned

**Figure 2:**
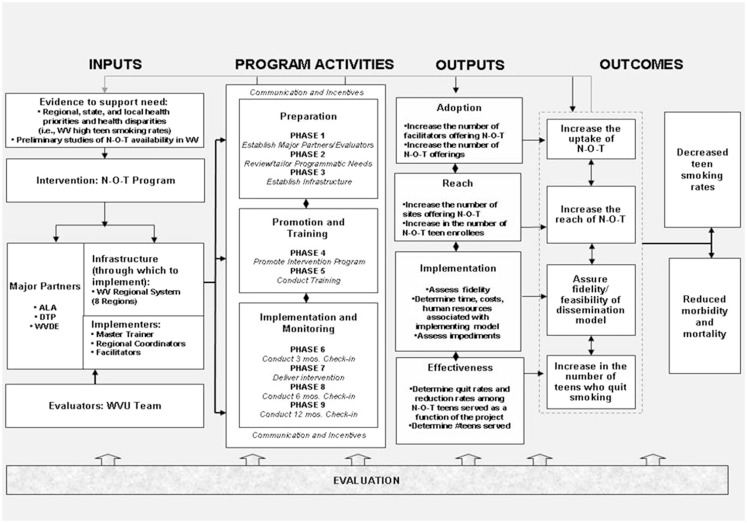
**Dissemination trial logic model using key components of REAIM**.

A handful of dissemination models exist, but rigorous trials testing dissemination research are not extensively published. Consequently, there is a limited literature on the research designs appropriate to examine the efficacy of the type of model described in this article. As a next step, we used the RE-AIM (37, 53) evaluation framework to *design* a randomized controlled trial to examine the following aspects of the dissemination model: reach into the target population; Effectiveness of the intended intervention; Adoption by target settings; Implementation and consistency of delivery; and Maintenance of use or effects over time (53). More specifically, reach is the absolute number/proportion of individuals who are willing to participate in a given intervention, in this case N-O-T. We will also examine reach across facilitators and teen participants. Adoption represents the absolute number and proportion of settings and facilitators who initiate the program as a function of the model implementation. Adoption, we will be measured by uptake. Implementation efficiency refers to the implementers’ adherence to the components of the dissemination model protocol and to the feasibility of such action. We will examine systems level fidelity and feasibility as a function of our model implementation. Effectiveness refers to the impact of an intervention or service on key health outcomes. We will examine efficacy at the N-O-T program level through determination of teen participants’ smoking outcomes. Of note, *Maintenance* is not a measurement focus at this early point in model implementation. As shown in Figure 2, specific outcomes in the context of RE-AIM will be captured as a function of our trial.

We are currently conducting this trial in WV, to be completed by the end of 2014. The logic model in Figure 2 provides an overview of the important facets of our current study, including inputs, program activities, outputs, and outcomes. The final outcomes will be presented upon trial completion. However, because the field of dissemination research is lacking in detailed methodologies for implementation, it is not premature to discuss the lessons learned thus far in our evaluation especially as they relate to partnerships, flexibility, and communication. Table 1 *highlights necessary components of dissemination, based the implementation of the nine phase model, including examples of challenges or successes at each phase*.

**Table 1:** **Application of model phases**.

Model phase	Critical component	Application examples
1. Establish major partners and evaluators	Involve stakeholders and intended adopters early in the planning and development process	Created methods of joint decision making across major partners
	Consider dissemination as a central component “intervention” development process	Sought ongoing partner insights about real world function and pre-load mechanisms to assist partners when things didn’t go as planned
	Major partners should have existing infrastructure that can aid dissemination efforts	
2. Review and tailor programmatic needs	Orient dissemination strategies toward the needs of the end users (e.g., align with needs assessment)	Used varied dissemination methods, including written information, electronic media, and person-to-person communication
	Minimize the extent to which adoption and implementation conflict with the economic or administrative incentives of the users or their communities	Used the flexibility of the model to address the pre-identified barriers with tailored solutions
3. Establish infrastructure	Develop a regional (or equivalent) model consistent with existing partner infrastructure	State-level: key partners were gatekeepers for access to sites that serve teens and expert staff in tobacco prevention and cessation
		Regional-level: created a regional system to provide primary points of contact across region to more evenly distribute the division of labor
		Site-level (school or community center): provided meeting space, mechanisms to recruit teens and access to facilitators
4. Promote intervention program	Draw upon existing resources, relationships, and networks while building new resources as needed	Changes in our Regional Coordinator staff created challenges with Phase 4 necessitating new Regional Coordinators via an alternative source (i.e., the Extension Service) – although challenging this allowed for new delivery sites
	Include both proactive and reactive dissemination channels (e.g., include information that users have identified as important and include information that users may not know to request but that they are likely to need)	
5. Conduct Regional Coordinator and facilitator training	Simplify and clearly state information so that the users can understand their specific and required practices or tasks, and roles/responsibilities	Both Regional Coordinator and facilitator training included hands on examples and easy to understand reporting for program delivery
	Make sure effort compensation is understood up front	
6. Conduct 3 month check in	Establish linkages between practitioners and researchers because the amount and quality of exchange between them are essential components of successful dissemination	Ongoing adjustments and improvements to communication among research staff, the state coordinator, Regional Coordinators, key partners, facilitators and delivery sites were essential to implementation
	Plan according to the five distinct steps in adoption as outlined by Diffusion of Innovations theory (knowledge, persuasion, decision, implementation and confirmation)	
	Establish linkages to external resources that may be needed to implement the interventions (technical assistance)	
7. Deliver intervention	Allow for flexibility to achieve balance between “fidelity” and “adaptation” of interventions and where delivery is optimal	Worked with all partners to operationalize the definitions of community and school sites
8. Conduct 6 month check in	Include effective quality control mechanisms to assure that system information is accurate/relevant; reinforce decisions to adopt and relevance to adopters (per Diffusion of Innovations theory and Social Cognitive Theory)	Planned check-ins between Coordinators and facilitators allowed research staff to identify and address challenges early, supply reinforcement of adoption utilizing modeling and incentives
9. Conduct 12 month check in	See Phase 8	See Phase 8

### Partnerships

Identification and engagement of key partners, including gate keepers, stakeholders, and intended adopters in the early phases of the development process are critical. We were fortunate to have established relationships with state leaders in public health and education, the ALA, and community members (via the WV PRC Community Partnership Board) who provided guidance in the model structure, potential incentives, and assisted in troubleshooting challenges as they arose. Specific to this project, these relationships were invaluable when a critical component of the model, the RTPS network, dissolved. Community and organization partners helped to identify a new and equally viable option, the WVU Extension Service. This change created delays specifically in Phase 4 of the model, but also allowed for new partnerships to emerge.

In applied science, particularly implementation and dissemination science, feedback from trusted partners (and partners who trust you) and those who will actually implement the program is critical to disseminate programs to scale. Although building relationships and trust takes time, in order to develop an intervention and dissemination plan, it is important to involve stakeholders and intended adopters early in the development process to provide feedback about viability, the socio-political context in which the intervention is to take place, work flow, resources, communication channels, and reporting (what and to whom).

Also, a lesson from this project that could be applied broadly to dissemination science is the critical link between researchers and practitioners. These linkages allow for honest feedback about what is working in the field and allow researchers to make timely adjustments to components of the model and assess progress. In addition, this project is unique in that the Regional and State Coordinators are not just program implementers, but also research participants. They, too, are providing data to assess the model. This distinctive relationship allowed Coordinators to provide feedback at the assigned checkpoints, but to also participate in research meetings. This relationship was carefully navigated at the beginning of the project, but proved invaluable as the need to self-reflect and make adjustments allowed the project to move forward.

### Flexibility

Although it is important to maintain consistency and model fidelity when testing a dissemination model for effectiveness, it is equally important to understand that in real world settings, events happen that may alter the best laid plans. Within the first 3 years of this project, there were multiple staff changes at each level of the model. This was a major challenge that required time for each of the new staff to learn their roles and develop the relationships discussed above. In the field, these staffing challenges primarily affected Phase 4 of the model, “Promote the Intervention,” because each new staff member had to reestablish community connections in order to create buy-in for the intervention.

Changing political environments, increased demands on school personnel time, and health priorities in schools away from tobacco interventions created situations in which the traditional model of delivery for the not-on-tobacco program were more difficult. In order to address these changes, this model explored alternative delivery methods and new partnerships, specifically in community organization settings that serve youth. This flexibility allowed for the program to grow beyond the school setting and created opportunities for new and important partnerships that did not exist previously. We also decided to expand the program to facilitators in the treatment regions who were previously trained by the WV ALA. To reduce burden on these currently trained facilitators, research staff worked with the State Coordinator and Regional Coordinators to create an Online training module and consent form for those who wanted to participate in the research study.

As with many programs over the last decade, this project faced funding challenges that required adjustments in scope. Because of delays due to noted infrastructure challenges, funding cut backs, and staffing challenges, the scope of the program was scaled back to incorporate two treatment regions with which we could work closely and really try to understand the mechanisms that led to barriers and successes within the model structure.

The built-in feedback loops in the model allowed for Regional Coordinators and the State Coordinator to provide insight into recruitment strategies in the field that were not necessarily envisioned by research staff. This included recruitment materials such as posters and pamphlets, channels such as social media and committee meetings, and potential facilitators from community organizations that were unknown to research staff during the planning process (Phases 1 and 4). In addition to the reactive dissemination channels described above, research staff provided proactive information that users may not know to request but that they are likely to need. They also indicated that the incentives built-in to the model were not sufficient to overcome some of the same challenges identified to facilitators in the needs assessment phase of the model, the most prominent of which remained, lack of time to implement the program.

### Communication

The final lesson learned thus far is the critical importance of consistent communication among all levels of the research team. Consistent meetings of a core research team allowed for timely decisions and protocol adjustments as challenges arose. In addition, the research team asked for assistance, insights, and recommendations from the WV Prevention Research Center (PRC) Community Partnership Board throughout the life of the project.

A major challenge with the current model was embedded in the nature of the additional scope of work required by our WVU Extension Service Regional Coordinators. Our Extension partners were *out in the field* much of the time. Because they serve very rural areas of the state of WV, they often did not have cell service or access to the Internet, making communication and reporting difficult. However, the built-in check-ins often corrected for this and allowed for communication among research staff, the State Coordinator, and Regional Coordinators.

It was critical throughout this process to be in constant communication with key partners identified both in Phase 1, and new partners identified in Phases 3 and 4 to provide updates, solicit feedback about potential adjustments to the protocols, and identify and address barriers to success. Finally, research staff kept the funding agency apprised of all changes throughout the process to ensure that the goals of the project were fulfilled to their satisfaction.

## Conclusion

The present model is among the first field-tested dissemination models of an evidence-based teen smoking cessation intervention. The nine-phase model has a sound theoretical foundation utilizing critical constructs in intervention diffusion, health behavior, and social marketing. The development and first years of implementation illuminated key lessons that are applicable beyond teen smoking cessation programs. Although the final outcomes of this model are not known, several of the key lessons can be applied to dissemination science beyond a teen smoking cessation programing. A main focus of the present model is to de-centralize responsibility and allow for a local locus of control (i.e., Regional Coordinators). The intent is to better meet the needs and challenges of N-O-T facilitators and teens at the local level, while maintaining fidelity to an evidence-based program statewide. In this WV example, this de-centralization used state-level coordination to guide and support the new structure while working to eliminate some of the burden of recruitment, communication, and record keeping associated with not-on-tobacco program evaluation locally. It also served to create new partnerships and highlighted the need for flexibility and multiple channels and levels of communication across numerous stakeholders.

## Author Contributions

Kimberly Horn conceived the study, drafted the majority of the manuscript, and provided critical content review and final revisions. Traci Jarrett also made substantial contributions to the manuscript and implemented the study. Andrew Anesetti-Rothermel and Nancy O’Hara Tompkins assisted in data interpretation and provided revisions. Geri Dino made substantial contributions to the conception and design of the study.

## Conflict of Interest Statement

The authors declare that the research was conducted in the absence of any commercial or financial relationships that could be construed as a potential conflict of interest. Authors Horn and Dino are co-developers of N-O-T; however, the American Lung Association owns all copyrights and the developers have no rights to ownership and receive no financial remuneration for program use or distribution.
